# Proteome Profiling Uncovers an Autoimmune Response Signature That Reflects Ovarian Cancer Pathogenesis

**DOI:** 10.3390/cancers12020485

**Published:** 2020-02-19

**Authors:** Makoto Kobayashi, Hiroyuki Katayama, Ehsan Irajizad, Jody V. Vykoukal, Johannes F. Fahrmann, Deepali L. Kundnani, Chuan-Yih Yu, Yining Cai, Fu Chung Hsiao, Wei-Lei Yang, Zhen Lu, Joseph Celestino, James P. Long, Kim-Ann Do, Karen H. Lu, Jon J. Ladd, Nicole Urban, Robert C. Bast Jr., Samir M. Hanash

**Affiliations:** 1 Department of Clinical Cancer Prevention, The University of Texas M.D. Anderson Cancer Center, 1515 Holcombe Blvd, Houston, TX 77030, USA; dm11018s@st.kitasato-u.ac.jp (M.K.); hkatayama1@mdanderson.org (H.K.); jvykouka@mdanderson.org (J.V.V.); jffahrmann@mdanderson.org (J.F.F.); deepali.kundnani@gatech.edu (D.L.K.); cyu3@mdanderson.org (C.-Y.Y.); ycai4@mdanderson.org (Y.C.); fchsiao@mdanderson.org (F.C.H.); 2 Department of Biostatistics, The University of Texas M.D. Anderson Cancer Center, 1515 Holcombe Blvd, Houston, TX 77030, USA; eirajizad@mdanderson.org (E.I.); jplong@mdanderson.org (J.P.L.); kimdo@mdanderson.org (K.-A.D.); 3 Department of Experimental Therapeutics, The University of Texas M.D. Anderson Cancer Center, 1515 Holcombe Blvd, Houston, TX 77030, USA; newworldyang@gmail.com (W.-L.Y.); zlu@mdanderson.org (Z.L.); rbast@mdanderson.org (R.C.B.J.); 4 Department of Gynecologic Oncology and Reproductive Medicine, The University of Texas M.D. Anderson Cancer Center, 1515 Holcombe Blvd, Houston, TX 77030, USA; jcelesti@mdanderson.org (J.C.); khlu@mdanderson.org (K.H.L.); 5 Translational Research Program, Public Health Sciences, Fred Hutchinson Cancer Research Center, 1100 Fairview Ave N, Seattle, WA 98109, USA; jladd@fredhutch.org (J.J.L.); nurban@fredhutch.org (N.U.)

**Keywords:** ovarian cancer, autoantibody signature, TP53–MYC network, antigen–antibody complexes

## Abstract

Harnessing the immune response to tumor antigens in the form of autoantibodies, which occurs early during tumor development, has relevance to the detection of cancer at early stages. We conducted an initial screen of antigens associated with an autoantibody response in serous ovarian cancer using recombinant protein arrays. The top 25 recombinants that exhibited increased reactivity with cases compared to controls revealed TP53 and MYC, which are ovarian cancer driver genes, as major network nodes. A mass spectrometry based independent analysis of circulating immunoglobulin (Ig)-bound proteins in ovarian cancer and of ovarian cancer cell surface MHC-II bound peptides also revealed a TP53–MYC related network of antigens. Our findings support the occurrence of a humoral immune response to antigens linked to ovarian cancer driver genes that may have utility for early detection applications.

## 1. Introduction

A humoral immune response in the form of autoantibodies to tumor antigens occurs early during tumor development. Identification of antigens that induce a selective autoantibody response associated with a particular cancer type has translational relevance for cancer screening [1,2,3]. There is currently an ongoing search for biomarkers that have utility for ovarian cancer early detection. The overall five-year survival rate for this cancer is below 30%, as over 70% of patients are diagnosed with stages III or IV disease. However, subjects diagnosed with localized disease have a survival rate of 75–90% [4]. At present, cancer antigen 125 (CA125) is the most investigated early detection marker for ovarian cancer [5]. Sequential monitoring of subjects with ultrasound and for elevated circulating levels of CA125 can achieve moderate specificity [6], but with limited sensitivity. There remains a need for identification of additional markers for ovarian cancer early detection. Tumor associated autoantibodies may improve on the performance of CA125 alone as we recently described for the human epididymis protein 4 (HE4) antigen–autoantibody complexes as complementing CA125 for detecting early-stage ovarian cancer [7].

Multiple approaches are currently available for the discovery of tumor antigens that induce a humoral autoantibody response. No single approach allows a comprehensive assessment of the full repertoire of epitopes associated with an autoantibody response in cancer. cDNA expression libraries [8], phage display [9] and recombinant protein arrays [10,11,12] have been utilized to identify antigens associated with autoantibodies. Other approaches include natural protein arrays that utilize fractionated tumor cell lysates as the source of antigens to preserve post-translational modifications (PTMs) and other protein alterations associated with immune reactivity [13,14,15]. Recently we have reported on the use of whole-genome derived peptide arrays as an approach for identification of pre-diagnostic autoantibodies associated with lung cancer, which provides a comprehensive coverage of peptide epitopes encoded in the genome [16].

In this study we explored the relationship of the autoantibody response in ovarian cancer to disease pathogenesis. We first investigated the repertoire of antigens that induce a humoral immune response in ovarian cancer using recombinant protein arrays, which was followed by analysis of circulating antigen–antibody complexes in ovarian cancer using mass spectrometry. We also profiled using mass spectrometry ovarian cancer cell line MHC-II bound peptides as a potential source of epitopes associated with autoantibodies. Integrated data analyses yielded immune network signatures involving TP53 and MYC, which are major contributors to the pathogenesis of ovarian cancer.

## 2. Results

### 2.1. Recombinant Protein Array-Based Ovarian Cancer Autoantibody Signature

**Figure 1:**
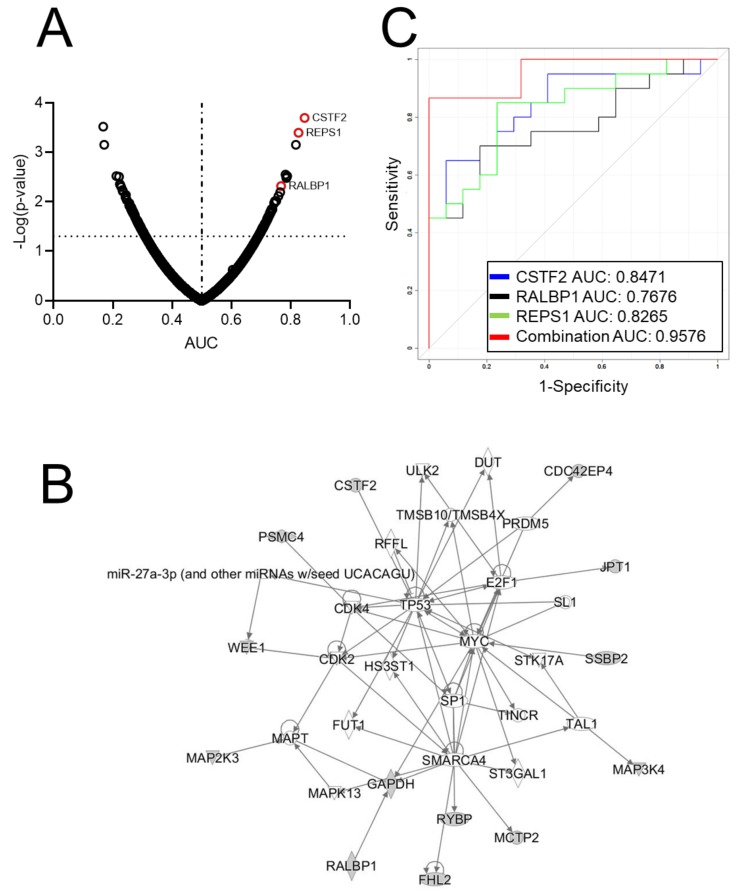
Ovarian cancer autoantibody signature based on recombinant protein array analysis. (**A**) Volcano plot illustrating the AUC (*x*-axis) and –log10 (*p*-value) (*y*-axis) distribution of autoantibodies against 5005 recombinant proteins. Dashed line indicates border line of significance (Y = 1.3) and solid line indicates AUC = 0.5. (**B**) Ingenuity Pathway Analysis (IPA) network based on the top 25 performing autoantibodies based on AUC point estimates. (**C**) Classification performance of autoantibodies against CSTF2, RALBP1, REPS1 and the combination of the three markers in distinguishing ovarian cases from controls.

We investigated the antibody reactivity of 20 serous ovarian cancer cases and 17 controls (Table S1) using recombinant protein arrays. The IgG reactivity of 75 recombinant proteins showed a statistically significant increase in ovarian cancer cases compared to controls (*p* < 0.05, Figure 1A and Table S2). Applying stricter criteria (*p* < 0.02, two-tailed Wilcoxon signed-rank test) narrowed the list to 25 recombinants (Table 1). The direct interaction network analysis using Ingenuity Pathway Analysis (IPA) for these top reactive proteins revealed TP53 and MYC to be the major central network nodes (Figure 1B).

The top performing proteins were cleavage stimulating factor 2 (CSTF2) (receiver operating characteristic area under the curve: AUC = 0.847, *p* = 0.0002) and RalA-binding protein 1 (RALBP1) (AUC = 0.768, *p* = 0.0048). Interestingly, RalBP1-associated Eps domain-containing protein 1 (REPS1), which was among the top reactive proteins (AUC = 0.827, *p* = 0.0004), is known to associate with RALBP1 [17,18], suggestive of immunoglobulin (Ig) reactivity against two interacting proteins. We confirmed the direct interaction between these two proteins using the STRING database (https://string-db.org/) (Figure S1A) and by co-immunoprecipitation (Co-IP) using OVCAR8 and DOV13 cell lines (Figure S1B). The combined performance of these three markers resulted in an AUC of 0.9576 (Figure 1C).

### 2.2. Circulating Immunoglobulin (Ig)-Bound Protein Signature in Ovarian Cancer

**Figure 2:**
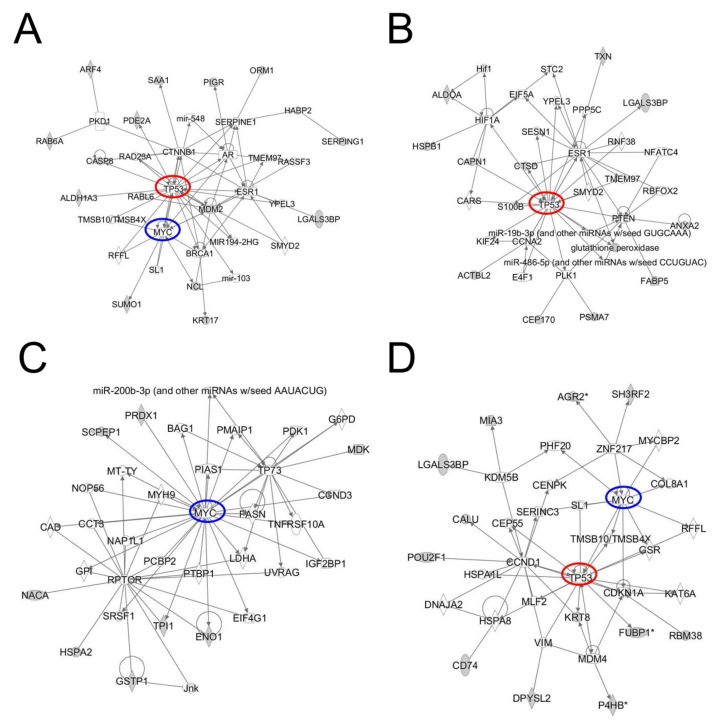
Autoimmune response signatures for ovarian cancer. Ingenuity Pathway Analysis (IPA) for immunoglobulin (Ig)-bound proteins that had a case–control ratio > 1.2 in early stage ovarian cancer and for which the protein target was identified ovarian cancer cell lines (**A**). (**B**) and (**C**) represent the top 2 IPA-derived networks based on Ig-bound protein features described in Table 3. (**D**) represents IPA-derived network based on OVCAR8 MHC-II bound peptidome. TP53 and MYC are marked as red and blue, respectively.

Released antigens may occur in circulation bound to Ig [15,19,20,21]. We profiled ovarian cancer circulating Ig-bound proteins in ovarian cancer subjects compared to controls using tandem mass tag (TMT)-based liquid chromatography mass spectrometry (LCMS). For TMT labeling experiments (see methods), three sample sets (sample set-1, -2 and -3) were prepared; each sample set consisted of four pooled case samples (each pool was comprised of three cases, *n *= 36 in total) and two pooled control samples (each pool was comprised of 10 age-matched healthy controls). Patient information is provided in Table S1. Pooling strategies were as follows: Case pools 1, 2, 5 and 6 consisted of CA125 negative (defined as < 35 U/mL) cases, case pools 3, 4, 7 and 8 consisted of CA125 positive (≥ 35 U/mL) cases and case pools 9–12 were based on histology (Table S1). We filtered out abundant plasma proteins as contaminants and considered proteins as tumor-derived antigen candidates using the following criteria: (i) case/control ratios of Ig-bound proteins greater than 1.2 identified in at least two sets, (ii) protein products of genes expressed in ovarian cancer cells [22], which yielded 24 proteins (Table 2). Interestingly, IPA again revealed the top protein network as centered around TP53, MYC and ESR1 (Figure 2A) with functions consisting of cell cycle, cell death and survival and organismal injury (Table S3).

We additionally performed Ig-bound protein analysis using pre-diagnostic samples consisting of four cases and 40 age matched control combined into two pools. A strict filtering criterion was applied to identify immunoglobulin (Ig)-bound protein targets that were (1) exclusively identified in plasma of cases and (2) the protein target was expressed in ovarian cancer cell lines [22], the results of which yielded 44 Ig-bound proteins (Table 3). Based on IPA, the top network represented TP53 (Figure 2B, Table S3), and the second network represented MYC (Figure 2C, Table S3). According to gene ontology (GO) analysis, the biological function of these peptides was related to leukocyte mediated immunity (*p* = 9.22 × 10^−22^, FDR = 1.47 × 10^−2^) and immune response (*p* = 1.22 × 10^−5^, FDR = 3.88 × 10^−2^) (Supplementary Table 4). Raw data is provided in Table S5.

### 2.3. Immunopeptidome Analysis

MHC class I peptides are associated with a T-cell mediated immune response [23], whereas MHC class II peptides are associated with a humoral B-cell response [19]. Given our interest in identifying autoantibody signatures, we profiled OVCAR8 cell line MHC-II bound peptidome by LCMS to identify the repertoire of peptides that would potentially induce a B cell driven IgG autoantibody response. A total of 92 identified peptides met the criteria of 13–25 amino acid length as MHC-II bound peptides [24] (Table 4). IPA of the 92 peptides yielded a TP53 and MYC driven network (Figure 2D).

## 3. Discussion

Using two proteomics platforms with independent subject samples, we investigated autoimmune response networks of antigenic proteins and peptides in ovarian cancer. We observed in the initial discovery set significant reactivity against 75 recombinants with ovarian cancer sera compared to controls.

Notably, it has previously been reported that autoantibodies against RALBP1, transcriptional adapter 3 (TALD3L), E3 ubiquitin–protein ligase CBL-B (CBLB) and serine/arginine-rich splicing factor 10 (FUSIP1) are statistically significantly elevated in sera of ovarian cancer patients in comparison to healthy controls [10]. Consistently, our independent analysis also indicated elevated autoantibody reactivity against these protein targets with corresponding AUCs of 0.767, 0.685, 0.653 and 0.653, respectively, for delineating ovarian cancer cases from healthy controls. Thus, our findings and those of others were validated [10].

Using IPA, the 25 top performers in the current study were part of a TP53 and MYC network. Given prior publications of autoantibodies in ovarian cancer using different platforms to search for autoantibodies, we performed similar IPA on data from other reports to determine associated networks (Figure S2) [10,25,26,27].

Consistent with our findings, we uncovered TP53 and MYC as major nodes for antigens associated with autoantibodies, suggesting an intrinsic relationship between established drivers of serous ovarian cancer pathogenesis and autoantibody targets [28]. We previously reported on a triple-negative breast cancer (TNBC) autoimmune response signature that was also mainly contributed by TP53 and MYC [15]. According to the Cancer Genome Atlas (TCGA), serous ovarian carcinoma and the basal type of breast cancer have molecular phenotype similarity that include MYC high expression and high frequency of TP53 inactivation [29]. Thus, a similarity in driver genes would account for similarity in the autoimmune response network between the two cancer types.

Autoantibodies to TP53 itself are known to be elevated in various types of cancer [30,31,32,33]. Shimada et al. reported positivity of TP53 autoantibody was detected in about 20% of cancer patients [34]. In ovarian cancer, Yang et al. reported the utility of TP53 autoantibody for early ovarian cancer detection combined with CA125 based on pre-diagnostic samples [6]. Additionally, MYC autoantibodies have been reported in ovarian cancer [26,35,36]. These results suggested that gene alternations such as amplification or mutation will trigger TP53 and MYC autoantibody production in ovarian cancer. TP53 and MYC were not part of the set of recombinants on the array we have utilized. Similarly, mass spectrometry-based detection of TP53 and MYC is often lacking because of sensitivity and/or post-translational modifications [37].

CSTF2, RALBP1 and its binding partner REPS1 were part of the TP53 and MYC signature and showed significant performance with an AUC = 0.958. CSTF2 was a member of the cleavage stimulation factor (CSTF) complex that is involved in the 3’ end cleavage and in polyadenylation of pre-mRNAs [38]. Evidence suggests that regulation of polyadenylation may play an important role in cell growth control and tumor development [39]. The formation of a complex between CSTF, BARD1/BRCA1 and TP53 has been reported to repress mRNA polyadenylation following treatment of cells with DNA-damage-inducing agents, suggesting that CSTF may have a direct role in the development of ovarian cancer [40]. CSTF2 mRNA expression was low or absent in most normal tissues suggesting that the presence of autoantibodies to this protein was reflective of its dysregulated expression in ovarian cancer [41]. Moreover, RALBP1 binding was critical for the activation of Ral signaling in Ras-induced transformation and tumorigenesis of human cells [42]. Dysregulation of micro-143-3p and RALBP1 has been reported to contribute to the pathogenesis of ovarian cancer [43]. REPS1 is a binding partner of RALBP1 that was found to play a role in regulating EGF receptors and Ral-GTPases activity [17]. Collectively, these findings highlight that the target antigens identified in this study are related to the pathogenesis of ovarian cancer.

Mass spectrometry-based circulating Ig-bound protein analysis yielded concordant results with respect to TP53 and MYC driven network with both newly diagnosed and pre-diagnostic samples. Likewise, ovarian cancer cell surface MHC-II bound peptidome analysis showed clearly a TP53 and MYC centered signature. These results further reinforce the role of the driver genes TP53 and MYC in inducing proteins that trigger a humoral immune response.

There is increasing evidence for circulating immune complexes during tumor development that may serve as cancer biomarkers. We recently reported that human epididymis protein 4 (HE4) antigen–autoantibody complexes could significantly improve diagnostic performance in combination with CA125 compared with CA125 alone based on analysis of early stage ovarian cancer samples [7]. Other complexes notably involving cofilin 1 were found to be associated with pancreatic cancer [44].

We acknowledge that there is limited overlap between protein–autoantibody targets identified through the recombinant protein arrays with that of Ig-bound antigen complexes identified via mass spectrometry. There are a multitude of strategies available for discovery of tumor antigens directed autoantibodies in circulation. Each strategy targets a different repertoire of antigens and presents both advantages and disadvantages as we have previously outlined in a review [21]. The primary intent of this study is to explore the relationship of the autoantibody response in ovarian cancer to pathogenesis. Thus, we intentionally employed a multi-platform approach to uncover a diversity of autoantibodies with a goal to ascertain their relationship to disease pathogenesis given that different platforms would identify different autoantibodies but that may reflect the same underlying origin.

In conclusion, our data from this study as well as pathway analysis of other reported data is indicative of an autoimmune response targeting antigens regulated by driver genes in ovarian cancer. Further validation of autoantibodies against targets that exhibited high performance notably CSTF2, RALBP1 and REPS1 will be needed. If successful, such autoantibody targets may offer utility for early detection of ovarian cancer.

## 4. Materials and Methods

### 4.1. Recombinant Protein Array Analysis

For the autoantibody discovery analysis using recombinant protein arrays, blood samples were collected at the Fred Hutchinson Cancer Research Center following Institutional Review Board approval and informed consent (no ethic code and protocol numbers were assigned). The subjects were women diagnosed with serous ovarian cancer and controls consisting of apparently healthy women attending regular breast cancer screening exams and women undergoing gynecologic surgery for a variety of conditions but with normal ovarian pathology. Controls were matched to cases for age, race, family history of ovarian and breast cancer and collection date. Subject information is provided in Table S1.

Recombinant protein arrays containing 5005 recombinants arrayed in duplicate were utilized in the initial discovery phase (Thermo Fisher Scientific, Waltham, MA, USA). Alexa 647-labeled anti-human IgG (Thermo Fisher Scientific) was utilized for quantification of reactivity. Serum samples were assessed for IgG reactivity against arrayed proteins using a three-step indirect immunofluorescence protocol. All steps were done at 4 °C. Briefly, a blocking reaction for protein microarrays was done using a blocking buffer (PBS with 1% BSA and 0.1% Tween-20) for 1 h. Serum samples were diluted 1:150 in the probing/washing buffer (PBS with 1% BSA, 0.5 mM DTT, 5 mM MgCl_2_, 0.05% TritonX-100 and 5% glycerol) and applied onto the microarrays and incubated for 2.5 h. Following washing with the washing buffer for 3 × 10 min, microarrays were incubated with 1 µg/mL Alexa 647-labeled anti-human IgG antibody diluted in the washing buffer for 1 h. The washing buffer was subsequently applied for 3 × 10 min, followed by drying via spinning at 500 × g for 2 min. All microarrays were scanned with a GenePix 4200A scanner using the same settings. Scanned images were analyzed using GenePix 6.0 microarray analysis software. Local background subtracted median spot intensities were used for downstream statistical analysis.

### 4.2. Analysis of Circulating Ig-Bound Proteins in Ovarian Cancer

For mass spectrometry based circulating Ig-bound protein analysis, blood samples from ovarian cancer patients and from healthy controls who did not develop ovarian cancer were collected at the University of Texas M.D. Anderson Cancer Center Gynecologic Tissue Bank. All samples were collected following Institutional Review Board approval and informed consent. Subject information is available in Table S1. The study cohort at MD Anderson is MDACC-NROSS. The protocol number of the study at MD Anderson is ID01-022.

Detailed information regarding mass spectrometry-based analysis of Ig-bound protein complexes is described elsewhere [19]. Briefly, Ig-bound proteins from a total of 100 μL of plasma were extracted using NAb protein A/G spin columns (Thermo Fisher Scientific) according to the manufacturer’s instructions. Columns were equilibrated twice with 400 μL binding buffer (phosphate buffered saline; PBS, pH 7.2) and then incubated for 10 min at room temperature (RT) with plasma samples diluted 1:2 in PBS, pH 7.2. Columns were washed three times with 400 μL of PBS, pH 7.2. Ig-bound proteins were eluted twice with 400 μL of 0.1 M glycine, pH 3. The flow-through was collected and then neutralized with 40 μL of PBS, pH 9. After each step, columns were centrifuged for 1 min at 5000× *g*. To reduce non-specific binding to the protein A/G spin columns, an additional low pH wash with 400 μL of PBS, pH 5, was performed before Ig-bound protein elution.

For mass spectrometry analysis, the collected proteins were treated with 25 mM TCEP for Cys reduction and subsequently alkylated with acrylamide. The samples were next fractionated at the protein level by reverse-phase chromatography followed by desalting for 5 min with 95% mobile phase A (0.1% TFA in 95% H2O). Proteins were eluted from the column and collected into 12 fractions, with a gradient elution that included an increase from 5% to 70% mobile phase B (0.1% TFA in 95% acetonitrile) over 25 min, 70% to 95% mobile phase B for 3 min, a wash step to hold at 95% mobile phase B for 2 min, followed by a re-equilibration step at 95% mobile phase A for 5 min.

### 4.3. Immunopeptidome Analysis

The OVCAR8 cell line was incubated with 50 ng/mL interferon gamma (IFN γ) for 24 h before collecting MHC-II bound peptides from 500 million cells. MHC-II bound peptides were eluted, processed and analyzed by LC-MS/MS and searched using our previously reported methodology [19,23].

### 4.4. Mass Spectrometry Analysis

For Ig-bound protein analysis, protein digestion and identification by LC-MS/MS was performed using our established protocol [19,45,46]. Briefly, a nanoAcquity UPLC system coupled in-line with WATERS SYNAPT G2-Si mass spectrometer was used for the separation of pooled digested protein fractions. The system was equipped with a Waters Symmetry C18 nanoAcquity trap-column (180 μm × 20 mm, 5 μm) and a Waters HSS-T3 C18 nanoAcquity analytical column (75 μm × 150 mm, 1.8 μm). Data were acquired in resolution mode with SYNAPT G2-Si using Waters Masslynx (version 4.1, SCN 851). The mass spectrometer was operated in V-mode with a typical resolving power of at least 20,000. All analyses were performed using positive mode ESI using a NanoLockSpray source. The lock mass channel was sampled every 60 s. Accurate mass LC-HDMSE data were collected in an alternating, low energy (MS) and high energy (MSE) mode of acquisition with mass scan range from m/z 50 to 1800. The spectral acquisition time in each mode was 1.0 s with a 0.1 s inter-scan delay. The acquired LC-HDMSE data were processed and searched against protein knowledge database (Uniprot and TruEMBL, 92,355 human protein sequences) through ProteinLynx Global Server (Version 3.0.2, Waters Company) with 4% FDR.

### 4.5. Immunoprecipitation (IP) and Western Blot Analysis

Two ovarian cancer cell lines (OVCAR8 and DOV13) were washed two times with PBS and treated with IP lysis buffer (Thermo Fisher Scientific) at 4 °C for 30 min. After centrifugation at 20,000× *g* for 30 min at 4 °C, the supernatant was collected for IP. To conjugate primary antibody, 2 ug of anti-RALBP1 antibody (clone 2A1, Abnova, Taipei, Taiwan), 2 uL of anti-REPS1 antibody (clone D6F4, Cell Signaling Technology, Danvers, MA, USA), 2 ug of mouse isotype control IgG (clone 20102, R&D Systems, Minneapolis, MN, USA) and 2 ug of rabbit isotype control IgG (clone DA1E, Cell Signaling Technology) were mixed with Dynabeads protein G (Thermo Fisher Scientific) for 30 min at room temperature. Following incubation with antibody–Dynabeads conjugate and 1 mg of cell lysate overnight at 4 °C, antibody–antigen complex was washed three times with PBS. Precipitated proteins were eluted using Laemmli’s buffer (Bio-rad, Hercules, CA, USA). Western blotting was performed as previously described [46].

### 4.6. Ingenuity Pathway Analysis (IPA)

IPA (Version 49309495, Qiagen, Hilden, Germany) was utilized for network signature analysis with the following settings: (1) direct relationships, (2) excluded endogenous chemicals, (3) number of molecules per network was 35 and networks per analysis was 25 and (4) relationships considered were those experimentally observed and human.

### 4.7. Statistical Analysis

Recombinant protein array data were normalized with quantile normalization, and intensity measures for duplicate spots were averaged. A two-tailed Wilcoxon signed-rank test was applied to each recombinant protein to compare differences in mean intensity between cases and controls. Receiver operating characteristic (ROC) curve analysis was performed to assess the performance of biomarker candidates in distinguishing cases from controls. Model building was based on a logistic regression model. The AUC of the derived panel was determined by using the empirical ROC estimator of the linear combination corresponding to the model. The standard error (S.E.) and the corresponding 95% confidence intervals presented for the performance of each biomarker or biomarker panel were based on the bootstrap procedure in which we re-sampled with replacement separately for the controls and the diseased 1000 bootstrap samples. ROC curves and model building was performed using R statistical software version 3.3.1.

## 5. Conclusions

Our proteomics based data from this study as well as pathway analysis of other reported data is indicative of an autoimmune response targeting antigens regulated by driver genes such as TP53 and MYC in ovarian cancer.

## Figures and Tables

**Table 1:** Reactivity summary of the 25 most reactive recombinants in high-density recombinant protein arrays.

Gene	Accession	AUC	Wilcox t (2-Sided)
CSTF2	NM_001325	0.8471	0.0002
REPS1	BC021211	0.8265	0.0004
EFCBP2	BC016979	0.8176	0.0007
SSBP2	BC017020	0.7824	0.0028
MAP2K3	NM_002756	0.7882	0.003
PSMC4	NM_153001	0.7868	0.0031
MGC4473	NM_080719	0.7853	0.0033
RALBP1	NM_006788	0.7676	0.0048
AMMECR1	BC060813	0.7647	0.0064
WAC	BC004258	0.7588	0.0077
DCX	NM_178152	0.7515	0.0096
HN1	NM_016185	0.7471	0.0097
RYBP	BC014959	0.7471	0.0097
C13orf3	BC048988	0.7441	0.0107
LOC51334	BC038838	0.7441	0.0107
FHL2	NM_001450	0.7382	0.0141
CLPP	NM_006012	0.7382	0.0141
KIAA0174	BC004359	0.7382	0.0141
WEE1	NM_003390	0.7353	0.0141
LOC199964	BC029775	0.7368	0.0148
GAPD	NM_002046	0.7324	0.0154
HIPK4	NM_144685	0.7324	0.0154
MCTP2	BC025708	0.7324	0.0167
CDC42EP4	BC010451	0.7294	0.0169
MAP3K4	NM_005922	0.7279	0.0189

**Table 2:** Ig-bound proteins in early stage ovarian cancer. Number indicates mass spectral counts.

Gene	Newly Diagnosis Early Stage plasma set 1						Newly Diagnosis Early Stage Plasma Set 2						Newly Diagnosis Early Stage Plasma Set 3					
	Case Pool 1	Case Pool 2	Case Pool 3	Case Pool 4	Control	Control	Case Pool 5	Case Pool 6	Case Pool 7	Case Pool 8	Control	Control	Case Pool 9	Case pool 10	Case Pool 11	Case Pool 12	Control	Control
ALDH1A3	0	3	0	0	0	0	0	0	0	0	0	0	0	3	0	0	0	0
AMPD3	0	0	0	0	0	0	0	0	1	0	0	0	0	0	6	0	0	0
ARF4	0	0	0	0	0	0	4	0	0	0	0	0	0	0	3	0	0	0
BSCL2	0	1	0	0	0	0	0	0	0	1	0	0	0	0	0	0	0	0
C1R	13	0	12	33	35	32	0	0	0	40	0	0	11	0	39	0	0	12
CASP8	0	6	0	0	0	0	0	0	0	0	0	0	0	0	1	0	0	0
CCDC53	0	0	0	0	0	0	0	1	0	0	0	0	0	2	0	0	0	0
EPS8L2	1	0	0	0	0	0	0	0	0	0	0	0	0	0	4	3	0	0
IGHV2-5	4	7	7	10	6	4	4	0	0	1	3	0	1	7	28	4	5	2
KRT17	35	0	0	0	11	1	33	0	0	0	0	0	121	23	53	291	143	344
LGALS3BP	32	12	22	17	20	14	17	0	19	18	11	8	10	32	56	61	37	9
MDM2	1	0	0	0	0	0	0	0	0	0	0	0	0	0	2	0	0	0
NCL	0	0	0	1	0	0	0	0	0	0	0	0	0	2	0	0	0	0
ORM1	15	0	22	19	1	15	24	24	29	44	21	19	38	28	84	29	28	45
PDE2A	2	1	0	0	0	0	0	0	0	0	1	0	0	0	3	0	0	0
PIGR	6	5	12	0	0	0	0	0	0	0	0	0	10	0	17	18	0	14
RAB6A	0	0	0	0	0	0	1	0	0	0	0	0	0	2	0	1	0	0
RWDD4	0	1	1	0	0	0	0	0	0	0	0	0	0	0	0	1	0	0
SAA1	2	0	2	3	0	3	0	0	0	3	0	0	0	0	21	10	0	0
SERPING1	48	9	45	44	0	38	18	0	5	23	26	13	10	8	52	41	0	15
SUMO1	0	0	1	0	0	0	0	2	0	0	0	0	0	0	0	0	0	0
TANGO6	0	0	0	0	0	0	1	0	0	0	0	0	0	0	0	1	0	0
TSTA3	0	1	0	0	0	0	0	0	1	0	0	0	0	0	0	0	0	0
VPS26B	1	0	0	0	0	0	0	0	0	0	1	0	0	1	0	0	0	0

**Table 3:** Ig-bound proteins in pre-diagnostic ovarian cancer. Number indicates mass spectral counts.

Gene	Pre-Diagnostic Ovarian Cancer Plasma set					
	Case 1	Case 2	Case 3	Case 4	Control Pool 1	Control Pool 2
ACTBL2	1	0	0	1	0	0
ALDOA	0	0	0	1	0	0
BLMH	0	0	0	1	0	0
CALML5	0	0	0	7	0	0
CANT1	1	0	0	0	0	0
CAPN1	0	0	1	0	0	0
CEP170	1	0	0	0	0	0
CTSD	0	0	0	4	0	0
DBI	0	0	1	0	0	0
DOHH	1	0	0	0	0	0
EEF1A1	5	0	0	1	0	0
ENO1	6	0	0	13	0	0
EPS8L2	0	0	4	0	0	0
FABP5	4	0	0	6	0	0
GLOD4	0	0	0	1	0	0
GSTP1	2	0	0	0	0	0
HMOX2	0	0	1	0	0	0
HSPA2	1	0	0	0	0	0
HSPA8	0	0	0	3	0	0
HSPB1	10	0	0	0	0	0
HSPH1	3	0	0	0	0	0
KRT7	0	0	0	6	0	0
KRT84	6	0	0	5	0	0
LARP7	0	1	0	0	0	0
LGALS3BP	0	0	7	0	0	0
LYPLA1	0	0	2	0	0	0
MANSC1	0	0	0	1	0	0
MDK	1	0	0	0	0	0
MLX	0	0	1	0	0	0
NACA	0	0	1	0	0	0
PKM	23	0	0	23	0	0
PNP	0	0	0	3	0	0
POTEF	3	0	0	0	0	0
PRDX1	0	0	0	6	0	0
PSMA7	0	0	0	1	0	0
SCPEP1	0	0	1	0	0	0
SERPING1	0	0	3	0	0	0
STRBP	1	0	0	0	0	0
TIMM50	0	0	1	0	0	0
TPI1	1	0	0	1	0	0
TUBA1B	10	0	0	0	0	0
TUBA3C	5	0	0	0	0	0
TXN	5	0	0	1	0	0
UBB	0	0	0	4	0	0

**Table 4:** MHC-II bound peptides from OVCAR8 cell line.

Sequence	Length	Gene
VNQRNRTYSSGSSGGSHPS	19	ABI2
VERGGVVTSNPLGF	14	ACADVL
IVNTARPDEKAIMT	14	ACTN4
IITAVNPATIGREKDME	17	AGGF1
VYETTDKHLSPDGQYVPRIM	20	AGR2
VYETTDKHLSPDGQYVPRIM	20	AGR2
DAQLDAYNARMDTS	14	ALYREF
GRAGSQGQPAPGGRP	15	AMT
IQRTPKIQVYSRHPAENGKSNF	22	B2M
LTTDEYDGHSTYPSHQYQ	18	BLVRB
VLSSGKFYGDEEKDKGLQTSQD	22	CALR
SSGKFYGDEEKDKGLQTSQDARF	23	CALR
SSGKFYGDEEKDKGLQTSQDARF	23	CALR
FVGSQATDFGEAL	13	CALU
LNDMTPPVNPSRE	13	CANX
KESKLPGDKGLVL	13	CANX
EDPSSGLGVTKQDLGPVPM	19	CD74
YGMDYATSKDAREPVVG	17	CHID1
SVYTTTRSHLGAENNID	17	CLPTM1L
SITSVTSSVVSTSSNSSDNAP	21	DOCK5
FQGTKAALAGGTTM	14	DPYSL2
VQALDDTERGSGGFGSTGKN	20	DUT
LKKFSYRNAKNDDL	14	ERAP2
AVTDFEPTQARMAF	14	ERAP2
RVPFRRNKEEDLQSTKEERF	20	ERLEC1
LHTKGALPLDTVTF	14	ERP29
VKFDTQYPYGEKQDE	15	ERP29
VAEVGISDYGDKLNM	15	ERP29
AQTSPQGMPQHPPAPQGQ	18	FUBP1
YAQTSPQGMPQHPPAPQGQ	19	FUBP1
YYAQTSPQGMPQHPPAPQGQ	20	FUBP1
DVGENNQGGKPLIM	14	GALNT3
AAIRQAAKNGATGVEL	16	GDE1
AQEVTYANLRPFEA	14	GGCX
EKLPGQGVHSQGQGPGANF	19	GLG1
LASPEYVNLPINGNGKQ	17	GSTP1
FLASPEYVNLPINGNGKQ	18	GSTP1
IKKIADDKYNDTF	13	HSP90B1
LHVTDTGVGMTREE	14	HSP90B1
LVKNLGTIAKSGTSE	15	HSP90B1
LHVTDTGVGMTREEL	15	HSP90B1
VKNLGTIAKSGTSEF	15	HSP90B1
FLNKMTEAQEDGQSTSEL	18	HSP90B1
PFKVVEKKTKPYIQ	14	HSPA5
IIANDQGNRITPSY	14	HSPA5
IIANDQGNRITPSY	14	HSPA5
IVLVGGSTRIPKIQQL	16	HSPA5
TKMKETAEAYLGKKVTHA	18	HSPA5
VDIGGGQTKTFAPEEISA	18	HSPA5
VDIGGGQTKTFAPEEISAM	19	HSPA5
VAYGAAVQAGVLSGDQDTGD	20	HSPA5
AQQPAESRVSGISM	14	HSPG2
LVETTSLPPRPETT	14	HSPG2
LKENERFFGDSAASM	15	HYOU1
TREVEEEPGIHSLKHNKRVL	20	HYOU1
SVVSRTDSPSPTVL	14	KCT2
RGGLGGGYGGASGMGGITA	19	KRT8
VTDSSWSARKSQL	13	LGALS3BP
VSGMQHPGSAGGVY	14	LMAN1
VSGMQHPGSAGGVY	14	LMAN1
ILDSEKTSETAAKGVNTGGREPNTM	25	MIA3
VVEKTAAARLPPSVS	15	MVB12A
PASFTKNYKPVVQTTGN	17	NCEH1
IVIAKMDSTANEVE	14	P4HB
IVIAKMDSTANEVE	14	P4HB
LEGKIKPHLMSQEL	14	P4HB
IVIAKMDSTANEVEA	15	P4HB
FRPSHLTNKFEDKT	14	PDIA3
FRPSHLTNKFEDKTVA	16	PDIA3
IHTNWTGHGGTVSSSSYNA	19	PGD
LEGKVLPGVDALSNI	15	PGK1
LAQHGSEYQSVKL	13	PLOD1
FTVASASGAASTTTTASKAQ	20	POU2F1
GLFGKTVPKTVDNF	14	PPIB
VSMANAGKDTNGSQF	15	PPIB
LQAGKKSLEDQVEM	14	PRKCSH
IELQAGKKSLEDQVEM	16	PRKCSH
VQYQAPQLQPDRMQ	14	RBM38
YTKLGNPTRSEDL	13	RPN1
AHLGGGSTSRATSFLL	16	RPN1
IVETVYTHVLHPYPTQITQSEKQF	24	RPN1
QIPPLVTTDCMIQDQGNASPRFIRC	25	SEC24D
VSTASGTQTVFPSK	14	SH3RF2
PSGYKGRDCEVSLDSCSSGP	20	SLIT1
SSLLRPQPEPQQE	13	TAPBP
AATPGLNGQMPAAQEG	16	TAPBP
AATPGLNGQMPAAQEGAVAF	20	TAPBP
AATPGLNGQMPAAQEGAVAF	20	TAPBP
VQAVSDPSSPQYGKY	15	TPP1
FGKQFLRQNTGDDQTS	16	TVP23C
FLDPSGKVHPEIINENGNPSYKYF	24	TXNDC12
FTHGIQSAAHFVM	13	TXNDC5

## Supplementary Materials

The following are available online at https://www.mdpi.com/2072-6694/12/2/485/s1, Figure S1: REPS1 and RALBP1 as binding proteins, Figure S2: IPA of reported ovarian cancer diagnostic panels, Table S1: Patient characteristics, Table S2: Autoantibody reactivity based on high-density recombinant protein arrays, Table S3: Summary of IPA signature functions, Table S4: Gene ontology analysis for early stage Ig-bound proteins, Table S5: Identified Ig-bound proteins in this study.
