# Supplementary material for: Proteome Profiling Uncovers an Autoimmune Response Signature That Reflects Ovarian Cancer Pathogenesis
Source: Cancers (Basel). 2020 Feb 19;12(2):485. doi: 10.3390/cancers12020485 (PMC7072578; doi:10.3390/cancers12020485)
Supplement: Supplementary file 1 [file cancers-12-00485-s001.zip › cancers-704174-final-supplementary/cancers-704174-final-supplementary.docx]

Supplementary Materials

Proteome Profiling Uncovers an Autoimmune Response Signature That Reflects Ovarian Cancer Pathogenesis

Makoto Kobayashi, Hiroyuki Katayama, Ehsan Irajizad, Jody V. Vykoukal, Johannes F. Fahrmann, Deepali L. Kundnani, Chuan-Yih Yu, Yining Cai, Fu Chung Hsiao, Wei-Lei Yang, Zhen Lu, Joseph Celestino, James P. Long, Kim-Ann Do, Karen H. Lu, Jon J. Ladd, Nicole Urban^5^, Robert C. Bast Jr, Samir M. Hanash


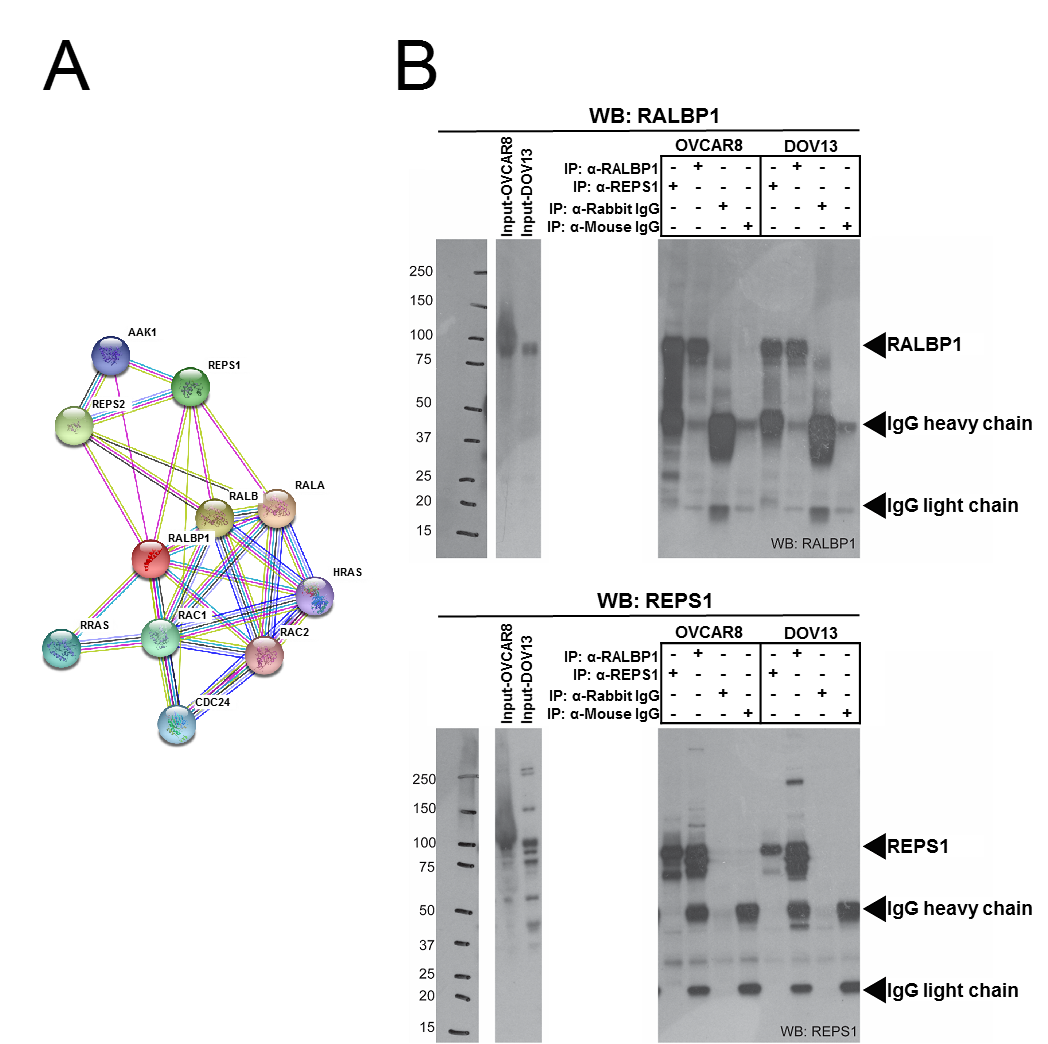


**Figure S1.** REPS1 and RALBP1 as binding proteins. (**A**) STRING image shows direct interaction between REPS1 and RALBP1 in human. (**B**) Co-immunoprecipitation (CO-IP) experiment using anti-RALBP1 and anti-REPS1 antibodies. Upper panel shows immunoblot for RALBP1 and lower panel shows immunoblot for REPS1.


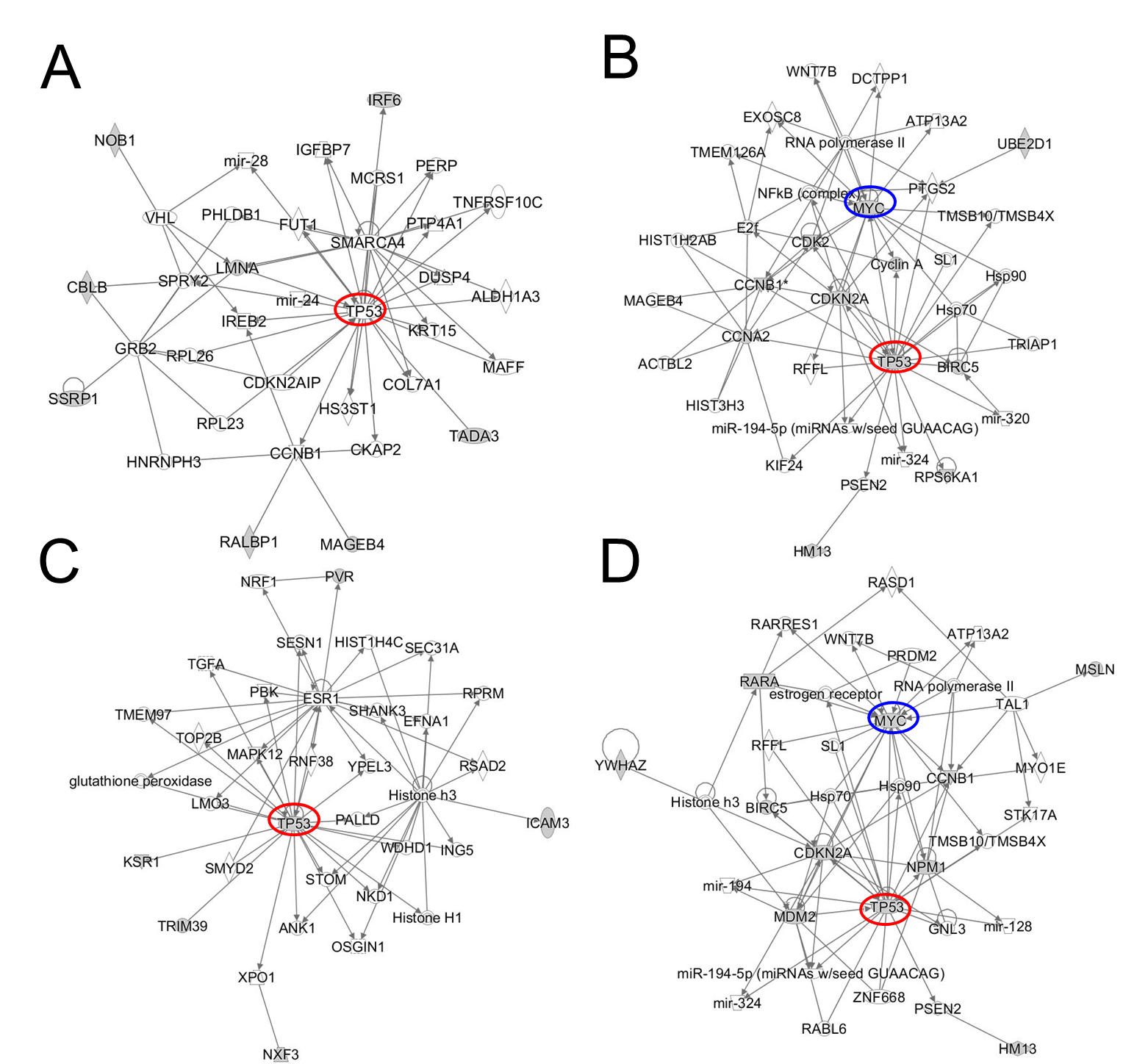


**Figure S2.** IPA of reported ovarian cancer diagnostic panels. (**A**) Top networks identified from 90 tumor-associated antigenic proteins reported by Hudson et al., based on IPA [1]. (**B**–**D**) Top network of ovarian cancer diagnostic autoantibody panel proteins reported by Li et al., [2], Katchman et al., [3] and Sun et al., [4].

**Table S1.** Patient characteristics.

**Autoantibody profiling by recombinant protein arrays.**

| Case, Control | Stage | Age | Histology | Differentiation | Risk status |
| --- | --- | --- | --- | --- | --- |
| Case | IC | 51 | High-grade serous ovarian carcinoma | Poorly differentiated | High |
| Case | IIIC | 58 | High-grade serous ovarian carcinoma | Moderately differentiated | Average |
| Case | IIIC | 70 | High-grade serous ovarian carcinoma | Moderately differentiated | Average |
| Case | IIA | 53 | High-grade serous ovarian carcinoma | Poorly differentiated | Average |
| Case | IIIB | 67 | High-grade serous ovarian carcinoma | Poorly differentiated | Average |
| Case | IVA | 71 | High-grade serous ovarian carcinoma | Poorly differentiated | Average |
| Case | IVA | 54 | High-grade serous ovarian carcinoma | Well differentiated | Average |
| Case | IVA | 50 | High-grade serous ovarian carcinoma | Poorly differentiated | High |
| Case | IIC | 65 | High-grade serous ovarian carcinoma | n/a | Average |
| Case | IIIC | 62 | High-grade serous ovarian carcinoma | n/a | Average |
| Case | IA | 52 | High-grade serous ovarian carcinoma | n/a | Average |
| Case | IC | 70 | High-grade serous ovarian carcinoma | n/a | Average |
| Case | IIIC | 59 | High-grade serous ovarian carcinoma | n/a | High |
| Case | IC | 61 | High-grade serous ovarian carcinoma | n/a | Average |
| Case | IIIC | 53 | High-grade serous ovarian carcinoma | n/a | High |
| Case | IIIC | 52 | High-grade serous ovarian carcinoma | n/a | Average |
| Case | IIIC | 74 | High-grade serous ovarian carcinoma | n/a | High |
| Case | IVB | 58 | High-grade serous ovarian carcinoma | n/a | Average |
| Case | IIIC | 71 | High-grade serous ovarian carcinoma | n/a | High |
| Case | IA | 58 | High-grade serous ovarian carcinoma | n/a | Average |
| Control | n/a | 62 | n/a | n/a | Average |
| Control | n/a | 65 | n/a | n/a | Average |
| Control | n/a | 52 | n/a | n/a | Average |
| Control | n/a | 69 | n/a | n/a | Average |
| Control | n/a | 58 | n/a | n/a | High |
| Control | n/a | 58 | n/a | n/a | High |
| Control | n/a | 52 | n/a | n/a | High |
| Control | n/a | 61 | n/a | n/a | High |
| Control | n/a | 62 | n/a | n/a | High |
| Control | n/a | 52 | n/a | n/a | Average |
| Control | n/a | 52 | n/a | n/a | Average |
| Control | n/a | 70 | n/a | n/a | Average |
| Control | n/a | 50 | n/a | n/a | Average |
| Control | n/a | 68 | n/a | n/a | Average |
| Control | n/a | 60 | n/a | n/a | Average |
| Control | n/a | 54 | n/a | n/a | Average |
| Control | n/a | 70 | n/a | n/a | Average |

**Samples for circulating Ig-bound protein analysis by mass spectrometry.**

| Case, Control | Stage | Age | Histology | CA125 (U/mL) |
| --- | --- | --- | --- | --- |
| Case pool 1 | I | 57 | High Grade Serous Carcinoma | 10.2 |
| Case pool 1 | IC | 77 | High Grade Serous Carcinoma | 11 |
| Case pool 1 | IA | 45 | Clear Cell Carcinoma | 12.2 |
| Case pool 2 | I | 67 | Endometrioid Adenocarcinoma | 18.2 |
| Case pool 2 | IC | 59 | Endometrioid Adenocarcinoma | 21.4 |
| Case pool 2 | I | 52 | Mucinous Adenocarcinoma | 26.3 |
| Case pool 3 | IC | 58 | Endometrioid Adenocarcinoma | 42.9 |
| Case pool 3 | IC | 70 | Endometrioid Adenocarcinoma | 45.9 |
| Case pool 3 | IA | 56 | Endometrioid Adenocarcinoma | 71.4 |
| Case pool 4 | IIC | 64 | High Grade Serous Carcinoma | 520.4 |
| Case pool 4 | IA | 54 | High Grade Serous Carcinoma | 899 |
| Case pool 4 | IC | 58 | High Grade Serous Carcinoma | 1354.8 |
| Case pool 5 | I | 41 | High Grade Serous Carcinoma | 13.24 |
| Case pool 5 | II | 59 | High Grade Serous Carcinoma | 24 |
| Case pool 5 | IA | 68 | Endometrioid Adenocarcinoma | 28.3 |
| Case pool 6 | I | 47 | Endometrioid Adenocarcinoma | 15.9 |
| Case pool 6 | I | 40 | Clear Cell Carcinoma | 18.8 |
| Case pool 6 | I | 18 | Endometrioid Adenocarcinoma | 20.7 |
| Case pool 7 | II | 61 | High Grade Serous Carcinoma | 81.9 |
| Case pool 7 | IA | 63 | High Grade Serous Carcinoma | 91.5 |
| Case pool 7 | II | 72 | High Grade Serous Carcinoma | 309.7 |
| Case pool 8 | IIA | 57 | High Grade Serous Carcinoma | 341.5 |
| Case pool 8 | IC | 84 | High Grade Serous Carcinoma | 421 |
| Case pool 8 | IIB | 49 | High Grade Serous Carcinoma | 1146 |
| Case pool 9 | IA | 62 | High Grade Serous Carcinoma | 24.9 |
| Case pool 9 | II | 62 | High Grade Serous Carcinoma | 54.6 |
| Case pool 9 | IIB | 79 | Low Grade Serous Carcinoma | 118.6 |
| Case pool 10 | IC | 69 | Endometrioid Adenocarcinoma | 73.1 |
| Case pool 10 | I | 53 | Endometrioid Adenocarcinoma | 410.4 |
| Case pool 10 | IC | 44 | Endometrioid Adenocarcinoma | 671 |
| Case pool 11 | I | 57 | Mucinous Adenocarcinoma | 12.5 |
| Case pool 11 | IA | 60 | Mucinous Adenocarcinoma | 112.9 |
| Case pool 11 | IA | 49 | mucinous adenocarcinoma | 120.9 |
| Case pool 12 | IIA | 49 | Clear Cell Carcinoma | 36.4 |
| Case pool 12 | IA | 65 | Clear Cell Carcinoma | 73.7 |
| Case pool 12 | IB | 46 | Clear Cell Carcinoma | 212.7 |
| Pre-diagnostic Case | I | 68 | Serous carcinoma of low malignant potential | 74.17 |
| Pre-diagnostic Case | I | 68 | Serous carcinoma of low malignant potential | 7.75 |
| Pre-diagnostic Case | IC | 57 | High grade carcinoma with Endometrioid +Clear cell carcinoma | 15.8 |
| Pre-diagnostic Case | IIb | 58 | High-grade serous and high-grade endometrioid carcinoma | 18.1 |

**Table S2.** Autoantibody reactivity based on high-density recombinant protein arrays.

| **Gene** | **Accsession** | **AUC** | **Wilcox t (2-sided)** |
| --- | --- | --- | --- |
| CSTF2 | NM_001325 | 0.8471 | 0.0002 |
| REPS1 | BC021211 | 0.8265 | 0.0004 |
| EFCBP2 | BC016979 | 0.8176 | 0.0007 |
| MAP2K3 | NM_002756 | 0.7882 | 0.003 |
| PSMC4 | NM_153001 | 0.7868 | 0.0031 |
| MGC4473 | NM_080719 | 0.7853 | 0.0033 |
| SSBP2 | BC017020 | 0.7824 | 0.0028 |
| RALBP1 | NM_006788 | 0.7676 | 0.0048 |
| AMMECR1 | BC060813 | 0.7647 | 0.0064 |
| WAC | BC004258 | 0.7588 | 0.0077 |
| DCX | NM_178152 | 0.7515 | 0.0096 |
| HN1 | NM_016185 | 0.7471 | 0.0097 |
| RYBP | BC014959 | 0.7471 | 0.0097 |
| C13orf3 | BC048988 | 0.7441 | 0.0107 |
| LOC51334 | BC038838 | 0.7441 | 0.0107 |
| FHL2 | NM_001450 | 0.7382 | 0.0141 |
| CLPP | NM_006012 | 0.7382 | 0.0141 |
| KIAA0174 | BC004359 | 0.7382 | 0.0141 |
| LOC199964 | BC029775 | 0.7368 | 0.0148 |
| WEE1 | NM_003390 | 0.7353 | 0.0141 |
| GAPD | NM_002046 | 0.7324 | 0.0154 |
| HIPK4 | NM_144685 | 0.7324 | 0.0154 |
| MCTP2 | BC025708 | 0.7324 | 0.0167 |
| CDC42EP4 | BC010451 | 0.7294 | 0.0169 |
| MAP3K4 | NM_005922 | 0.7279 | 0.0189 |
| MAP2K3 | NM_145109 | 0.7235 | 0.0201 |
| ZDHHC3 | BC015467 | 0.7235 | 0.0214 |
| GRK6 | NM_002082 | 0.7206 | 0.0219 |
| FLJ35107 | BC027729 | 0.7206 | 0.0219 |
| ANGPT1 | BC029406 | 0.7206 | 0.0232 |
| KCTD7 | BC042482 | 0.7191 | 0.0241 |
| C6orf145 | NM_183373 | 0.7176 | 0.0251 |
| CHC1 | NM_001269 | 0.7147 | 0.0271 |
| KIAA1193 | BC028203 | 0.7147 | 0.0271 |
| NRBF2 | BC011707 | 0.7132 | 0.0282 |
| PKM2 | BC035198 | 0.7118 | 0.0281 |
| OCLN | BC029886 | 0.7118 | 0.0293 |
| C1orf25 | BC045535 | 0.7103 | 0.0305 |
| RBMS3 | BC030290 | 0.7088 | 0.0316 |
| CXorf17 | NM_017848 | 0.7088 | 0.0316 |
| OR56B4 | XM_115092 | 0.7074 | 0.0328 |
| POLR2C | NM_002694 | 0.7074 | 0.0328 |
| BC029609 | BC029609 | 0.7074 | 0.0329 |
| TXNDC5 | NM_022085 | 0.7074 | 0.0329 |
| MGC20533 | BC010176 | 0.7074 | 0.0329 |
| LZTFL1 | NM_020347 | 0.7059 | 0.033 |
| TFF2 | NM_005423 | 0.7059 | 0.033 |
| BC021561 | BC021561 | 0.7059 | 0.033 |
| ZFYVE27 | NM_144588 | 0.7059 | 0.033 |
| PAK1 | NM_002576 | 0.7059 | 0.0341 |
| SARG | NM_023938 | 0.7059 | 0.0341 |
| SCML4 | NM_198081 | 0.7059 | 0.0342 |
| ZFP36 | NM_003407 | 0.7059 | 0.0342 |
| SAMSN1 | BC029112 | 0.7029 | 0.0357 |
| C14orf1 | NM_007176 | 0.7029 | 0.0357 |
| FLJ14346 | BC018206 | 0.7029 | 0.0368 |
| DKFZP564D172 | NM_032042 | 0.7029 | 0.0368 |
| FBF1 | BC023549 | 0.7 | 0.0386 |
| ZKSCAN1 | BC022378 | 0.7 | 0.0386 |
| FAM50A | NM_004699 | 0.7 | 0.0397 |
| C20orf18 | NM_031227 | 0.7 | 0.0397 |
| MGC21675 | NM_052861 | 0.6985 | 0.0411 |
| RAB4A | NM_004578 | 0.6985 | 0.0411 |
| LIN7B | NM_022165 | 0.6985 | 0.0411 |
| GPAA1 | NM_003801 | 0.6971 | 0.0426 |
| LOC51760 | BC004518 | 0.6971 | 0.0426 |
| GLO1 | BC001741 | 0.6971 | 0.0427 |
| DFFA | NM_004401 | 0.6971 | 0.0427 |
| EIF2B5 | NM_003907 | 0.6956 | 0.0443 |
| NCOA4 | NM_005437 | 0.6956 | 0.0443 |
| FLJ31528 | NM_144679 | 0.6941 | 0.045 |
| MAP3K5 | NM_005923 | 0.6941 | 0.0457 |
| C9orf86 | BC002945 | 0.6926 | 0.0476 |
| RPS6KC1 | NM_012424 | 0.6912 | 0.0485 |
| SFN | BC001550 | 0.6912 | 0.0493 |

**Table S3.** Summary of IPA signature functions.

| **Figure** | **Signature** | **Major node in signature** | **Rank of network** | **IPA signature function** |
| --- | --- | --- | --- | --- |
| Figure 1B | High-density recombinant protein array | TP53-MYC | 1 | **Cell cycle**, Cellular development, Cellular growth and proliferation |
| Figure 2A | Ig-bound protein (Early stage) | TP53-MYC | 1 | Cancer, **Cell cycle**, Cell death and survival |
| Figure 2B | Ig-bound protein (Pre-diagnostic) | TP53 | 1 | Cellular development, Cellular growth and proliferation, Connective tissue development and fucntion |
| Figure 2C | Ig-bound protein (Pre-diagnostic) | MYC | 2 | Cancer, Carbohydrate metabolism, cell death and survival |
| Figure 2D | OVCAR8 MHC-classII peptidome | TP53-MYC | 1 | **Cell cycle**, Lymphoid tissue structure and development, Tissue morphology |
| Supplementary figure 2A | Hudson et al. | TP53 | 1 | Cancer, **Cell cycle**, Connective tissue development and fucntion |
| Supplementary figure 2B | Li et al. | TP53-MYC | 1 | **Cell cycle**, Cellular compromise, DNA replication, recombination, and repair |
| Supplementary figure 2C | Katchman et al. | TP53-ESR1 | 1 | Cancer, Endocrine system disorders, Organism injury and abnormalities |
| Supplementary figure 2D | Sun et al. | TP53-MYC | 1 | **Cell cycle**, Cellular development, Cellular growth and proliferation |

**Table S4.** Gene ontology analysis for early stage Ig-bound proteins.

| **GO biological process complete** | **Fold Enrichment** | **Raw P-value** | **FDR** |
| --- | --- | --- | --- |
| leukocyte mediated immunity (GO:0002443) | 9.61 | 9.22E-07 | 1.47E-02 |
| response to stimulus (GO:0050896) | 2.15 | 5.77E-06 | 4.59E-02 |
| immune system process (GO:0002376) | 3.9 | 1.06E-05 | 5.61E-02 |
| immune effector process (GO:0002252) | 6.77 | 1.22E-05 | 4.84E-02 |
| immune response (GO:0006955) | 4.89 | 1.22E-05 | 3.88E-02 |
| vesicle-mediated transport (GO:0016192) | 4.67 | 1.81E-05 | 4.80E-02 |

Table S5: Please view at the excel file.

**References.**

1. Hudson, M.E.; Pozdnyakova, I.; Haines, K.; Mor, G.; Snyder, M. Identification of differentially expressed proteins in ovarian cancer using high-density protein microarrays. *Proc. Natl. Acad. Sci. U. S. A.* **2007**, *104*, 17494-17499, doi:10.1073/pnas.0708572104.

2. Li, L.; Wang, K.; Dai, L.; Wang, P.; Peng, X.X.; Zhang, J.Y. Detection of autoantibodies to multiple tumor-associated antigens in the immunodiagnosis of ovarian cancer. *Mol Med Rep* **2008**, *1*, 589-594.

3. Katchman, B.A.; Chowell, D.; Wallstrom, G.; Vitonis, A.F.; LaBaer, J.; Cramer, D.W.; Anderson, K.S. Autoantibody biomarkers for the detection of serous ovarian cancer. *Gynecol. Oncol.* **2017**, *146*, 129-136, doi:10.1016/j.ygyno.2017.04.005.

4. Sun, H.; Shi, J.X.; Zhang, H.F.; Xing, M.T.; Li, P.; Dai, L.P.; Luo, C.L.; Wang, X.; Wang, P.; Ye, H., et al. Serum autoantibodies against a panel of 15 tumor-associated antigens in the detection of ovarian cancer. *Tumour Biol.* **2017**, *39*, 1010428317699132, doi:10.1177/1010428317699132.

| 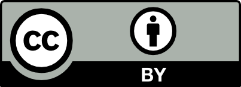 | © 2020 by the authors. Licensee MDPI, Basel, Switzerland. This article is an open access article distributed under the terms and conditions of the Creative Commons Attribution (CC BY) license (http://creativecommons.org/licenses/by/4.0/). |
| --- | --- |
